# Tetra-μ_3_-iodido-tetra­kis­{[ethyl 2-(1*H*-benzimidazol-1-yl)acetate-κ*N* ^3^]copper(I)}

**DOI:** 10.1107/S160053681202257X

**Published:** 2012-05-26

**Authors:** Lili Yang, Zhengyi Zhang

**Affiliations:** a Department of General Medicine, Lanzhou University Second Hospital, Lanzhou 730030, People’s Republic of China

## Abstract

The complex mol­ecule of the tetra­nuclear cubane-type title compound, [Cu_4_I_4_(C_11_H_12_N_2_O_2_)_4_], has crystallographically imposed fourfold inversion symmetry. The Cu^I^ ions are coordinated in a distorted tetra­hedral geometry by an N atom of a benzimidazole ring system and three μ_3_-iodide ions, forming a Cu_4_I_4_ core. In the crystal, complex mol­ecules are connected into a three-dimensional network by C—H⋯O hydrogen bonds involving H and O atoms of adjacent eth­oxy­carbonyl groups.

## Related literature

For potential applications in physiological and pharmacological fields of benzimidazoyl derivatives or complexes based on the benzimidazoyl unit, see: Ramla *et al.* (2007 ▶); Barreca *et al.* (2007 ▶); Cetinkaya *et al.* (1999 ▶); Snyderwine *et al.* (1997 ▶); Skog & Solyakov (2002 ▶); Garner *et al.* (1999 ▶). For applications of copper complexes in biology or medicine, see: Sorrell 1989 ▶. For related structures, see: Sun *et al.* (2011 ▶); Liu *et al.* (2011 ▶); Toth *et al.* (1987 ▶).
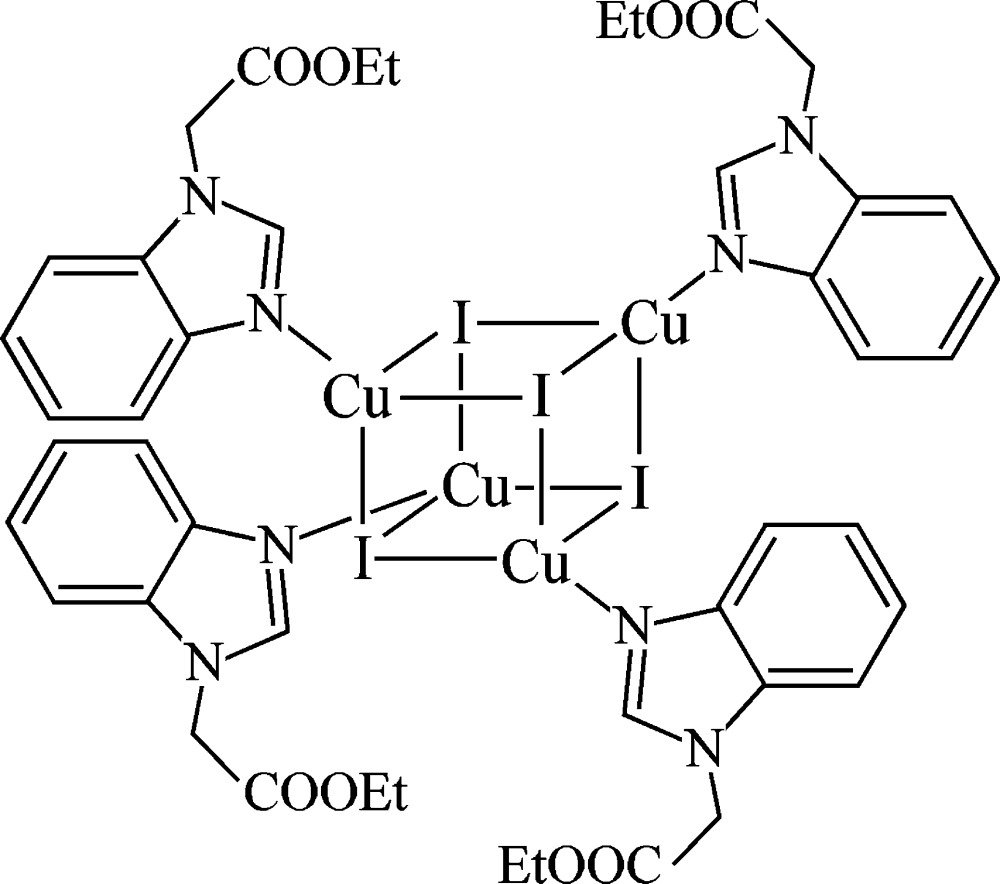


## Experimental

### 

#### Crystal data

[Cu_4_I_4_(C_11_H_12_N_2_O_2_)_4_]
*M* *_r_* = 1578.66
Tetragonal, 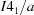

*a* = 21.196 (11) Å
*c* = 11.581 (7) Å
*V* = 5203 (5) Å^3^
*Z* = 4
Mo *K*α radiation
μ = 4.04 mm^−1^
*T* = 296 K
0.24 × 0.22 × 0.18 mm

#### Data collection

Bruker APEXII CCD diffractometer
Absorption correction: multi-scan (*SADABS*; Bruker, 2007 ▶) *T*_min_ = 0.768, *T*_max_ = 0.784
13042 measured reflections
2422 independent reflections
2018 reflections with *I* > 2σ(*I*)
*R*_int_ = 0.046

#### Refinement

*R*[*F* ^2^ > 2σ(*F* ^2^)] = 0.023
*wR*(*F* ^2^) = 0.053
*S* = 1.03
2422 reflections
155 parameters
H-atom parameters constrained
Δρ_max_ = 0.35 e Å^−3^
Δρ_min_ = −0.32 e Å^−3^

### 

Data collection: *APEX2* (Bruker, 2007 ▶); cell refinement: *SAINT* (Bruker, 2007 ▶); data reduction: *SAINT*; program(s) used to solve structure: *SHELXS97* (Sheldrick, 2008 ▶); program(s) used to refine structure: *SHELXL97* (Sheldrick, 2008 ▶); molecular graphics: *SHELXTL* (Sheldrick, 2008 ▶); software used to prepare material for publication: *SHELXTL*.

## Supplementary Material

Crystal structure: contains datablock(s) I, global. DOI: 10.1107/S160053681202257X/rz2757sup1.cif

Structure factors: contains datablock(s) I. DOI: 10.1107/S160053681202257X/rz2757Isup2.hkl

Additional supplementary materials:  crystallographic information; 3D view; checkCIF report

## Figures and Tables

**Table 1:** Hydrogen-bond geometry (Å, °)

*D*—H⋯*A*	*D*—H	H⋯*A*	*D*⋯*A*	*D*—H⋯*A*
C10—H10*A*⋯O1^i^	0.97	2.60	3.531 (5)	162

Symmetry code: (i) 
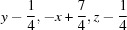
.

**Fig. 1.**
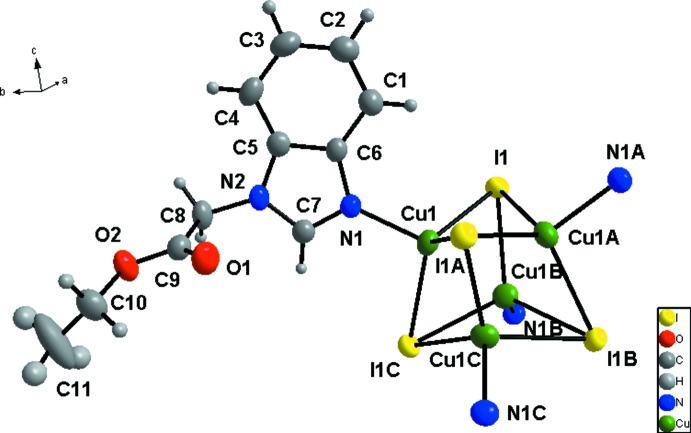
The structure of the title complex with displacement ellipsoids drawn at the 30% probability level. Atoms labelled with suffix A, B and C are generated by the symmetry operator (2-x, 1.5-y, z), (0.25+y, 1.75-x, 0.75-z) and (1.75-y, -0.25+x, 0.75-z) respectively.

**Fig. 2.**
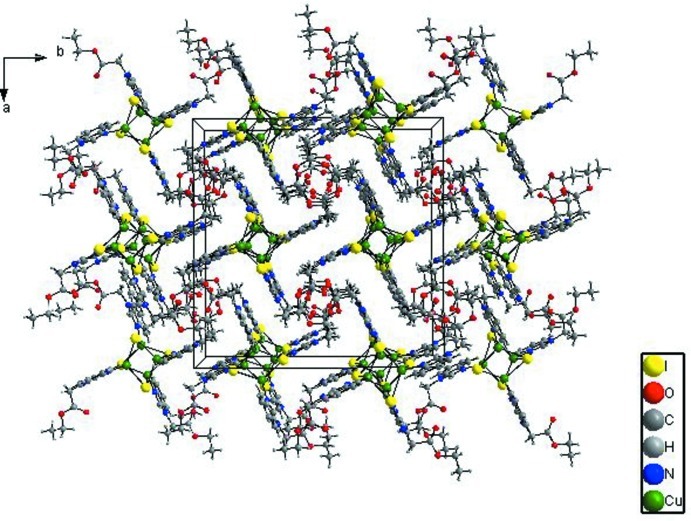
Packing diagram of the title compound viewed along the c axis.

## supplementary crystallographic information

### Comment

Benzimidazoyl-based ligands have wide applications in physiological and pharmacological fields, such as treatment of hypoglycemia, inhibitory activity for the lymphoma of Burkitt, antimicrobial activity and other effects (Ramla *et al.*, 2007; Barreca *et al.*, 2007; Cetinkaya *et al.*, 1999). Similarly, some metal complexes of benzimidazoyl derivatives possess interesting activities such as anti-viral, anti-cancers and anti-fungal activities (Snyderwine *et al.*, 1997; Skog & Solyakov,2002; Garner *et al.*, 1999). In particular, copper complexes are often used as chemical models of copper proteins and copper enzymes (Sorrell, 1989). Up to now, a number of structures of copper complexes involving the benzimidazol group have been reported (Sun *et al.*, 2011; Liu *et al.*, 2011; Toth *et al.*, 1987), but no crystal structure of copper(I) complex based on ethyl 2-(1*H*- benzimidazol-1-yl)acetate is available. In order to contribute to this research field, we report herein the crystal structure of the title tetranuclear cubane-type complex.

In the title complex (Fig. 1), each copper(I) metal of the Cu_4_I_4_ core is coordinated by three *µ_3_*-iodide ions and a nitrogen atom of a benzimidazole ring system in a distorted tetrahedral geometry. The deviation from the ideal geometry can be indicated by the range [104.30 (9)–114.311 (17) °] of the bond angles around the metal. The Cu—N bond length is 2.038 (3) Å, and the Cu—I bond lengths fall in the range are 2.715 (14)–2.733 (14) %A. In the crystal structure (Fig. 2), complex molecules are connected into a three-dimensional network by C—H···O hydrogen bonds (Table 1) involving H and O atoms of adjacent ethyl acetate groups.

### Experimental

A mixture of ethyl 2-(1*H*-benzimidazol-1-yl)acetate (0.015 g, 0.1 mmol), CuI (0.019 g, 0.1 mmol), 3 mL H_2_O and 10 mL EtOH was heated at 160°C under hydrothermal condition in a Teflon lined steel autoclave (inner volume 15 mL) for 3 days, and then cooled to room temperature at a rate of 2°C h^-1^. Red single crystals suitable for X-Ray diffraction were obtained in 43% yield. Elemental Calc. for C_44_H_48_Cu_4_I_4_N_8_O_8_: C, 33.48; H, 3.06; N, 7.10%. Found: C, 33.61; H, 3.21; N, 7.21%.

### Refinement

Carbon-bound H-atoms were placed in calculated positions (C—H = 0.93 Å) and were included in the refinement in the riding model approximation. The *U*_iso_(H) were allowed at 1.2 *U*_eq_ (C).

### Crystal data

**Table:**

[Cu_4_I_4_(C_11_H_12_N_2_O_2_)_4_]	*D*_x_ = 2.015 Mg m^−^^3^
*M**_r_* = 1578.66	Mo *K*α radiation, λ = 0.71073 Å
Tetragonal, *I*4_1_/*a*	Cell parameters from 4922 reflections
Hall symbol: -I 4ad	θ = 2.7–27.9°
*a* = 21.196 (11) Å	µ = 4.04 mm^−^^1^
*c* = 11.581 (7) Å	*T* = 296 K
*V* = 5203 (5) Å^3^	Block, red
*Z* = 4	0.24 × 0.22 × 0.18 mm
*F*(000) = 3040	

### Data collection

**Table:**

Bruker APEXII CCD diffractometer	2422 independent reflections
Radiation source: fine-focus sealed tube	2018 reflections with *I* > 2σ(*I*)
Graphite monochromator	*R*_int_ = 0.046
φ and ω scans	θ_max_ = 25.5°, θ_min_ = 1.9°
Absorption correction: multi-scan (*SADABS*; Bruker, 2007)	*h* = −14→25
*T*_min_ = 0.768, *T*_max_ = 0.784	*k* = −25→24
13042 measured reflections	*l* = −13→14

### Refinement

**Table:**

Refinement on *F*^2^	Primary atom site location: structure-invariant direct methods
Least-squares matrix: full	Secondary atom site location: difference Fourier map
*R*[*F*^2^ > 2σ(*F*^2^)] = 0.023	Hydrogen site location: inferred from neighbouring sites
*wR*(*F*^2^) = 0.053	H-atom parameters constrained
*S* = 1.03	*w* = 1/[σ^2^(*F*_o_^2^) + (0.0216*P*)^2^ + 3.0196*P*] where *P* = (*F*_o_^2^ + 2*F*_c_^2^)/3
2422 reflections	(Δ/σ)_max_ = 0.004
155 parameters	Δρ_max_ = 0.35 e Å^−^^3^
0 restraints	Δρ_min_ = −0.32 e Å^−^^3^

### Special details

**Table:**

Geometry. All e.s.d.'s (except the e.s.d. in the dihedral angle between two l.s. planes) are estimated using the full covariance matrix. The cell e.s.d.'s are taken into account individually in the estimation of e.s.d.'s in distances, angles and torsion angles; correlations between e.s.d.'s in cell parameters are only used when they are defined by crystal symmetry. An approximate (isotropic) treatment of cell e.s.d.'s is used for estimating e.s.d.'s involving l.s. planes.
Refinement. Refinement of *F*^2^ against ALL reflections. The weighted *R*-factor *wR* and goodness of fit *S* are based on *F*^2^, conventional *R*-factors *R* are based on *F*, with *F* set to zero for negative *F*^2^. The threshold expression of *F*^2^ > σ(*F*^2^) is used only for calculating *R*-factors(gt) *etc*. and is not relevant to the choice of reflections for refinement. *R*-factors based on *F*^2^ are statistically about twice as large as those based on *F*, and *R*- factors based on ALL data will be even larger.

### Fractional atomic coordinates and isotropic or equivalent isotropic displacement parameters (Å^2^)

**Table:**

	*x*	*y*	*z*	*U*_iso_*/*U*_eq_	
I1	1.102882 (10)	0.771393 (11)	0.517267 (18)	0.04331 (9)	
O2	0.78785 (12)	1.08340 (12)	0.5233 (2)	0.0575 (7)	
C9	0.81754 (17)	1.02891 (18)	0.5439 (3)	0.0471 (9)	
C11	0.7103 (2)	1.1347 (3)	0.4120 (7)	0.143 (3)	
H11A	0.7103	1.1719	0.4593	0.214*	
H11B	0.6699	1.1302	0.3753	0.214*	
H11C	0.7425	1.1383	0.3541	0.214*	
C8	0.88524 (16)	1.04277 (16)	0.5783 (3)	0.0506 (9)	
H8A	0.9052	1.0679	0.5186	0.061*	
H8B	0.8855	1.0671	0.6493	0.061*	
C10	0.72251 (18)	1.0806 (2)	0.4824 (4)	0.0664 (11)	
H10A	0.7160	1.0424	0.4379	0.080*	
H10B	0.6939	1.0800	0.5477	0.080*	
O1	0.79545 (12)	0.97762 (12)	0.5333 (2)	0.0598 (7)	
N2	0.92099 (13)	0.98507 (12)	0.5952 (2)	0.0443 (7)	
N1	0.96375 (13)	0.89215 (13)	0.5503 (2)	0.0435 (7)	
C7	0.93802 (16)	0.94424 (16)	0.5110 (3)	0.0440 (8)	
H7	0.9321	0.9523	0.4328	0.053*	
C5	0.93545 (16)	0.95618 (15)	0.7004 (3)	0.0428 (8)	
C6	0.96267 (15)	0.89832 (15)	0.6711 (3)	0.0405 (8)	
C4	0.92889 (18)	0.97605 (18)	0.8156 (3)	0.0556 (10)	
H4	0.9111	1.0148	0.8347	0.067*	
C3	0.95049 (19)	0.9346 (2)	0.8988 (3)	0.0626 (11)	
H3	0.9472	0.9457	0.9762	0.075*	
C1	0.98362 (18)	0.85733 (18)	0.7569 (3)	0.0538 (10)	
H1	1.0014	0.8185	0.7385	0.065*	
C2	0.97706 (18)	0.87645 (19)	0.8701 (3)	0.0603 (10)	
H2	0.9907	0.8499	0.9289	0.072*	
Cu1	0.98756 (2)	0.81434 (2)	0.45656 (4)	0.04928 (13)	

### Atomic displacement parameters (Å^2^)

**Table:**

	*U*^11^	*U*^22^	*U*^33^	*U*^12^	*U*^13^	*U*^23^
I1	0.03946 (14)	0.04686 (15)	0.04360 (13)	−0.00175 (11)	−0.00630 (9)	−0.00031 (10)
O2	0.0476 (15)	0.0452 (15)	0.0798 (18)	0.0054 (13)	−0.0103 (13)	0.0033 (13)
C9	0.047 (2)	0.047 (2)	0.0474 (19)	0.003 (2)	0.0016 (17)	−0.0051 (17)
C11	0.068 (3)	0.104 (4)	0.256 (9)	0.012 (4)	−0.033 (4)	0.080 (5)
C8	0.047 (2)	0.0350 (19)	0.070 (2)	0.0048 (18)	−0.0008 (18)	−0.0054 (17)
C10	0.050 (2)	0.074 (3)	0.076 (3)	0.004 (2)	−0.011 (2)	0.006 (2)
O1	0.0576 (17)	0.0428 (15)	0.0789 (18)	−0.0059 (14)	−0.0057 (14)	−0.0100 (13)
N2	0.0445 (17)	0.0343 (15)	0.0542 (17)	0.0042 (14)	−0.0006 (14)	−0.0027 (13)
N1	0.0467 (17)	0.0391 (16)	0.0446 (15)	0.0054 (14)	−0.0011 (13)	−0.0027 (13)
C7	0.042 (2)	0.043 (2)	0.0469 (19)	0.0004 (17)	−0.0031 (16)	−0.0032 (16)
C5	0.0369 (18)	0.042 (2)	0.049 (2)	−0.0038 (16)	0.0005 (16)	−0.0007 (16)
C6	0.0400 (19)	0.0360 (18)	0.0456 (18)	0.0006 (16)	0.0008 (15)	−0.0037 (15)
C4	0.057 (2)	0.053 (2)	0.058 (2)	0.002 (2)	0.0094 (19)	−0.0157 (19)
C3	0.071 (3)	0.074 (3)	0.043 (2)	−0.002 (2)	0.0065 (19)	−0.007 (2)
C1	0.062 (3)	0.044 (2)	0.055 (2)	0.003 (2)	−0.0007 (18)	0.0030 (17)
C2	0.068 (3)	0.062 (3)	0.051 (2)	−0.001 (2)	0.001 (2)	0.009 (2)
Cu1	0.0551 (3)	0.0414 (2)	0.0513 (3)	0.0049 (2)	0.0002 (2)	−0.0078 (2)

### Geometric parameters (Å, º)

**Table:**

I1—Cu1	2.7015 (14)	N1—C6	1.406 (4)
I1—Cu1^i^	2.7244 (16)	N1—Cu1	2.038 (3)
I1—Cu1^ii^	2.7334 (14)	C7—H7	0.9300
O2—C9	1.337 (4)	C5—C6	1.397 (4)
O2—C10	1.465 (4)	C5—C4	1.406 (5)
C9—O1	1.190 (4)	C6—C1	1.392 (5)
C9—C8	1.518 (5)	C4—C3	1.383 (5)
C11—C10	1.432 (6)	C4—H4	0.9300
C11—H11A	0.9600	C3—C2	1.395 (5)
C11—H11B	0.9600	C3—H3	0.9300
C11—H11C	0.9600	C1—C2	1.379 (5)
C10—H10A	0.9700	C1—H1	0.9300
C10—H10B	0.9700	C2—H2	0.9300
C8—N2	1.452 (4)	Cu1—I1^iii^	2.7244 (16)
C8—H8A	0.9700	Cu1—Cu1^iii^	2.7252 (13)
C8—H8B	0.9700	Cu1—Cu1^i^	2.7252 (13)
N2—C7	1.353 (4)	Cu1—I1^ii^	2.7334 (14)
N2—C5	1.397 (4)	Cu1—Cu1^ii^	2.7780 (17)
N1—C7	1.313 (4)		
			
Cu1—I1—Cu1^i^	60.299 (15)	C1—C6—C5	120.5 (3)
Cu1—I1—Cu1^ii^	61.477 (17)	C1—C6—N1	130.3 (3)
Cu1^i^—I1—Cu1^ii^	59.912 (15)	C5—C6—N1	109.3 (3)
C9—O2—C10	117.9 (3)	C3—C4—C5	116.0 (3)
O1—C9—O2	125.9 (3)	C3—C4—H4	122.0
O1—C9—C8	125.1 (3)	C5—C4—H4	122.0
O2—C9—C8	108.9 (3)	C4—C3—C2	122.0 (4)
C10—C11—H11A	109.5	C4—C3—H3	119.0
C10—C11—H11B	109.5	C2—C3—H3	119.0
H11A—C11—H11B	109.5	C2—C1—C6	117.5 (3)
C10—C11—H11C	109.5	C2—C1—H1	121.2
H11A—C11—H11C	109.5	C6—C1—H1	121.2
H11B—C11—H11C	109.5	C1—C2—C3	121.8 (4)
N2—C8—C9	111.5 (3)	C1—C2—H2	119.1
N2—C8—H8A	109.3	C3—C2—H2	119.1
C9—C8—H8A	109.3	N1—Cu1—I1	110.98 (8)
N2—C8—H8B	109.3	N1—Cu1—I1^iii^	104.30 (9)
C9—C8—H8B	109.3	I1—Cu1—I1^iii^	114.311 (17)
H8A—C8—H8B	108.0	N1—Cu1—Cu1^iii^	138.82 (8)
C11—C10—O2	108.8 (4)	I1—Cu1—Cu1^iii^	110.146 (17)
C11—C10—H10A	109.9	I1^iii^—Cu1—Cu1^iii^	59.43 (4)
O2—C10—H10A	109.9	N1—Cu1—Cu1^i^	147.54 (8)
C11—C10—H10B	109.9	I1—Cu1—Cu1^i^	60.27 (4)
O2—C10—H10B	109.9	I1^iii^—Cu1—Cu1^i^	60.21 (4)
H10A—C10—H10B	108.3	Cu1^iii^—Cu1—Cu1^i^	61.29 (3)
C7—N2—C5	106.8 (3)	N1—Cu1—I1^ii^	103.14 (8)
C7—N2—C8	125.5 (3)	I1—Cu1—I1^ii^	110.098 (19)
C5—N2—C8	126.9 (3)	I1^iii^—Cu1—I1^ii^	113.280 (17)
C7—N1—C6	105.1 (3)	Cu1^iii^—Cu1—I1^ii^	59.88 (4)
C7—N1—Cu1	126.8 (2)	Cu1^i^—Cu1—I1^ii^	109.193 (17)
C6—N1—Cu1	127.6 (2)	N1—Cu1—Cu1^ii^	147.30 (8)
N1—C7—N2	113.5 (3)	I1—Cu1—Cu1^ii^	59.827 (16)
N1—C7—H7	123.2	I1^iii^—Cu1—Cu1^ii^	107.917 (17)
N2—C7—H7	123.2	Cu1^iii^—Cu1—Cu1^ii^	59.357 (16)
C6—C5—N2	105.3 (3)	Cu1^i^—Cu1—Cu1^ii^	59.357 (16)
C6—C5—C4	122.3 (3)	I1^ii^—Cu1—Cu1^ii^	58.696 (16)
N2—C5—C4	132.4 (3)		
			
C10—O2—C9—O1	−0.9 (5)	C5—C6—C1—C2	−0.7 (5)
C10—O2—C9—C8	176.7 (3)	N1—C6—C1—C2	179.2 (3)
O1—C9—C8—N2	1.1 (5)	C6—C1—C2—C3	0.0 (6)
O2—C9—C8—N2	−176.6 (3)	C4—C3—C2—C1	0.4 (6)
C9—O2—C10—C11	−150.6 (4)	C7—N1—Cu1—I1	135.7 (3)
C9—C8—N2—C7	68.8 (4)	C6—N1—Cu1—I1	−54.4 (3)
C9—C8—N2—C5	−100.2 (4)	C7—N1—Cu1—I1^iii^	12.1 (3)
C6—N1—C7—N2	1.2 (4)	C6—N1—Cu1—I1^iii^	−177.9 (3)
Cu1—N1—C7—N2	173.0 (2)	C7—N1—Cu1—Cu1^iii^	−47.5 (3)
C5—N2—C7—N1	−1.7 (4)	C6—N1—Cu1—Cu1^iii^	122.5 (2)
C8—N2—C7—N1	−172.6 (3)	C7—N1—Cu1—Cu1^i^	68.4 (3)
C7—N2—C5—C6	1.5 (3)	C6—N1—Cu1—Cu1^i^	−121.6 (3)
C8—N2—C5—C6	172.1 (3)	C7—N1—Cu1—I1^ii^	−106.4 (3)
C7—N2—C5—C4	179.3 (4)	C6—N1—Cu1—I1^ii^	63.5 (3)
C8—N2—C5—C4	−10.0 (6)	C7—N1—Cu1—Cu1^ii^	−157.8 (2)
N2—C5—C6—C1	179.2 (3)	C6—N1—Cu1—Cu1^ii^	12.1 (4)
C4—C5—C6—C1	1.0 (5)	Cu1^i^—I1—Cu1—N1	−145.25 (9)
N2—C5—C6—N1	−0.8 (4)	Cu1^ii^—I1—Cu1—N1	145.04 (9)
C4—C5—C6—N1	−178.9 (3)	Cu1^i^—I1—Cu1—I1^iii^	−27.64 (3)
C7—N1—C6—C1	179.8 (4)	Cu1^ii^—I1—Cu1—I1^iii^	−97.34 (2)
Cu1—N1—C6—C1	8.2 (5)	Cu1^i^—I1—Cu1—Cu1^iii^	36.98 (3)
C7—N1—C6—C5	−0.2 (4)	Cu1^ii^—I1—Cu1—Cu1^iii^	−32.73 (3)
Cu1—N1—C6—C5	−171.9 (2)	Cu1^ii^—I1—Cu1—Cu1^i^	−69.703 (11)
C6—C5—C4—C3	−0.6 (5)	Cu1^i^—I1—Cu1—I1^ii^	101.19 (3)
N2—C5—C4—C3	−178.2 (3)	Cu1^ii^—I1—Cu1—I1^ii^	31.49 (3)
C5—C4—C3—C2	−0.1 (6)	Cu1^i^—I1—Cu1—Cu1^ii^	69.703 (11)

Symmetry codes: (i) *y*+1/4, −*x*+7/4, −*z*+3/4; (ii) −*x*+2, −*y*+3/2, *z*; (iii) −*y*+7/4, *x*−1/4, −*z*+3/4.

### Hydrogen-bond geometry (Å, º)

**Table:**

*D*—H···*A*	*D*—H	H···*A*	*D*···*A*	*D*—H···*A*
C10—H10*A*···O1^iv^	0.97	2.60	3.531 (5)	162

Symmetry code: (iv) *y*−1/4, −*x*+7/4, *z*−1/4.
